# Macrophage Plasticity and Function in the Lung Tumour Microenvironment Revealed in 3D Heterotypic Spheroid and Explant Models

**DOI:** 10.3390/biomedicines9030302

**Published:** 2021-03-15

**Authors:** Lauren Evans, Kate Milward, Richard Attanoos, Aled Clayton, Rachel Errington, Zsuzsanna Tabi

**Affiliations:** 1 Tissue MicroEnvironment Group, Division of Cancer and Genetics, School of Medicine, Cardiff University, Tenovus Building, University Hospital of Wales, Heath Park, Cardiff CF14 4XN, UK; Claytona@cardiff.ac.uk (A.C.); Erringtonrj@cardiff.ac.uk (R.E.); Tabiz@cardiff.ac.uk (Z.T.); 2 School of Medicine, Cardiff University, University Hospital of Wales, Heath Park, Cardiff CF14 4XN, UK; Richard.attanoos@wales.nhs.uk; 3 Department of Cellular Pathology, University Hospital of Wales, Heath Park, Cardiff CF14 4XN, UK

**Keywords:** heterotypic spheroids, myeloid cells, NSCLC, tumour explants, tumour microenvironment

## Abstract

In non-small cell lung cancer (NSCLC), stroma-resident and tumour-infiltrating macrophages may facilitate an immunosuppressive tumour microenvironment (TME) and hamper immunotherapeutic responses. Analysis of tumour-associated macrophage (TAM) plasticity in NSCLC is largely lacking. We established a novel, multi-marker, dual analysis approach for assessing monocyte-derived macrophage (Mφ) polarisation and M1/M2 phenotypic plasticity. We developed a flow cytometry-based, two-marker analysis (CD64 and CD206) of CD14^+^ cells. The phenotype and immune function of in vitro-induced TAMs was studied in a heterotypic spheroid and tumour-derived explant model of NSCLC. Heterotypic spheroids and NSCLC explants skewed Mφs from an M1- (CD206^lo^CD64^hi^) to M2-like (CD206^hi^CD64^lo^) phenotype. Lipopolysaccharide (LPS) and IFNγ treatment reversed M2-like Mφ polarisation, indicating the plasticity of Mφs. Importantly, antigen-specific CD8^+^ T cell responses were reduced in the presence of tumour explant-conditioned Mφs, but not spheroid-conditioned Mφs, suggesting explants are likely a more relevant model of the immune TME than cell line-derived spheroids. Our data indicates the importance of multi-marker, functional analyses within Mφ subsets and the advantages of the ex vivo NSCLC explant model in immunomodulation studies. We highlight the plasticity of the M1/M2 phenotype using the explant model and provide a tool for studying therapeutic interventions designed to reprogram M2-like Mφ-induced immunosuppression.

## 1. Introduction

Solid cancers comprise not only malignant cells, but also additional cancer-associated stromal and hematopoietic cells. Macrophages are the most abundant tumour-infiltrating immune cells and exist following the recruitment of blood monocytes or macrophages to the tumour tissue, where they differentiate into tumour-associated macrophages (TAMs) [1]. TAMs can promote or suppress anti-tumour immune responses, as discussed later. Whilst macrophages display high phenotypic plasticity, two major subsets have been described: the classically activated (M1) or the alternatively activated (M2) subset, based on exposure to different environmental stimuli [2,3]. M1-like macrophages are activated by Th1 cytokines, including IFNγ and TNFα, or Toll-like receptor ligands, namely lipopolysaccharide (LPS) [4], and have been shown to promote cytotoxic CD8^+^ T cell responses through production of pro-inflammatory cytokines (IL-6, IFNγ, TNFα, and inducible nitric oxide (iNOS)) [5]. In contrast, M2-like macrophage activation is induced by Th2 cytokines (IL-4, IL-10, IL-13, IL-1β) [4,5]. M2-like cells then promote inhibitory/regulatory T cell development through production of anti-inflammatory cytokines (TGFβ1, IL-10). In cancer, macrophage polarisation is influenced by cytokines and interactions with multicellular components in the tumour microenvironment (TME). Although both M1- and M2-like TAMs exist within the TME, in general, M2-like TAMs tend to be more frequent and have been more strongly associated with tumour progression and poor prognosis [2,4,6,7]. In NSCLC, TAMs are often broadly and unsatisfactorily defined by the expression of the pan-macrophage marker CD68 [8,9], or are poorly characterised by the single marker expression of M2-like markers such as CD23, CD206, and CD163, which heavily fluctuate with chemical stimuli and environmental influences [6,10]. Whilst the latter indicates the high sensitivity of these markers, the phenotypic plasticity and functional roles of TAMs in NSCLC are not well explored. Here, we established a novel two-marker approach to simultaneously detect M1- and M2-like TAMs within the lung TME, based on distinguishing CD206^lo^CD64^hi^ and CD206^hi^CD64^lo^ populations, respectively.

An immunosuppressive TME is a potent barrier to anti-cancer immunity, culminating in reduced therapeutic efficacy of both conventional and novel immunotherapeutic treatments for patients [11]. The lack of relevant models that genuinely reflect the multicellularity and heterogeneity of human tumours limits our ability to predict treatment efficacy [12]. Whilst advances in modelling have been made in recent years through the generation of 3D-spheroid and 3D-organoid models, as well as the development of better in vivo models, these systems are not without limitations. The homogenisation of tumour tissue into a cellular suspension prior to the formation of organoids means that the original structure and composition of the tissue is lost, whilst culturing conditions additionally impose selection pressures on the resultant 3D structures [13,14]. Heterotypic NSCLC spheroid models have been extremely useful for deciphering how chemotherapeutic and immune-modifying agents modulate different cellular compartments to influence the Mφ phenotype [15,16,17,18]. However, lack of functional data using these models limits the biological relevance of the findings. With regard to the in vivo expansion of tumour tissue within immune-deficient mice, the establishment and monitoring of these models require significant efforts and expenses, and most often are imperfect for human immunotherapeutic research [14]. As a result, all the above factors have been major driving forces for the development of alternative higher throughput tissue-modelling approaches which recapitulate the 3D architecture and complex cellular heterogeneity of the TME. These predominantly include ex vivo explant systems using primary tumour tissue that reflects an in situ patient TME that can be manipulated [19,20,21,22].

Here, we compare the phenotype and function of macrophages in a heterotypic spheroid and explant models of early stage NSCLC. We show that two-marker analyses of Fc-γ receptor (CD64) and mannose receptor (CD206) expression on tumour-conditioned monocyte-derived macrophages (Mφs) could concurrently identify M1- and M2-like Mφ populations by flow cytometry through assessment of distinct, CD206^lo^CD64^hi^ and CD206^hi^CD64^lo^ populations of CD14^+^ cells, respectively. With the use of this novel two-marker approach we demonstrate that human myeloid cells are polarised into an M2-like Mφ phenotype following conditioning with either tumour spheroids or explants. Importantly, we show that heterotypic spheroids are not functionally immunosuppressive, and fail to influence T cell activity despite inducing an M2-like Mφ phenotype. In contrast, tumour explant-conditioned Mφs recapitulate similar phenotypic M2 polarisation, yet are able to strongly suppress CD8^+^ T cell activity. Our work demonstrates the importance of assessing both macrophage phenotype and function, and highlights that a conventional 3D spheroid approach may not mimic the complexity of the TME required to achieve this.

## 2. Materials and Methods

### 2.1. Cell Lines

H522 cell line were purchased from ATCC (ATCC CRL-5810; Virginia, United States) and primary fibroblasts (AG02603) purchased from Coriell Institute for Medical Research (New Jersey, United States) and used at passages 8 to 12. All cultures were maintained in complete medium composed of RPMI-1640 (Lonza, Switzerland) supplemented with 10% fetal bovine serum (FBS; Thermo Fisher Scientific, Massachusetts, United States), 1 nM sodium-pyruvate (Sigma-Aldrich; Missouri, United States), 25 nM HEPES (Sigma-Aldrich; Missouri, United States), 100 U/mL penicillin (Lonza, Switzerland), 100 μg/mL streptomycin (Lonza, Switzerland), and 2 nM L-glutamine (Thermo Fisher Scientific, Massachusetts, United States). Cell lines were mycoplasma-free and tested regularly.

### 2.2. CD14^+^ Cell Isolation and Monocyte-Derived Macrophage Culture

Venous blood was collected from healthy donors (Cardiff University School of Medicine Ethics Committee, project approval number 18/17; 19/04/2018) or NSCLC patients (Wales Cancer Bank) [23], project approval number 17/016; 25/08/2017) under informed consent and with ethical approval according to the Helsinki Declaration and institutional standards. Peripheral blood mononuclear cells (PBMCs) were isolated by density gradient centrifugation on Histopaque (Sigma-Aldrich; Missouri, United States). Myeloid cells were separated by positive selection, using the EasySep^®^ Human CD14 Positive Selection Kit II (StemCell Technologies; Vancouver, Canada), in accordance with manufacturer’s instructions. Average CD14^+^ purity following positive selection was 95%, as measured by flow cytometry. Myeloid cells were cultured for 48 h in complete medium containing polarising cytokines to generate Mφs M1-like Mφs were generated through treatment with 20 ng/mL IFNγ (PBL Assay Science; New Jersey, United States) and 10 pg/mL LPS (Sigma-Aldrich; Missouri, United States), and M2-like Mφs through treatment with 20 ng/mL IL-4 (PeproTech; New Jersey, United States), 20 ng/mL IL-13 (PeproTech; New Jersey, United States), and 10 ng/mL IL-10 (R&D Systems; Minnesota, United States). Monocytes were cultured without added cytokines to generate unpolarised, media control Mφs.

For functional T cell experiments, CD14^+^ PBMCs were isolated by positive selection as described above. The negatively selected portion containing the CD14^−^ PBMCs underwent a further wash and magnetic separation step to reduce CD14^−^ cell contamination (0.9%) and increase CD3^+^ cell purity (67%). The resultant negatively selected CD14^−^ PBMCs were stored at −80 °C until used as the autologous T cell-containing population in functional experiments.

### 2.3. Multi-Component Spheroid Culture

Spheroids were generated via the cell aggregation (or pellet culture) method, as described previously [24,25]. In brief, to generate homotypic or heterotypic spheroids, H522 tumour cells and AG02603 fibroblasts (AGFB) were seeded alone or together at a ratio of 4:1 (tumour: fibroblast; 1 × 10^4^ total cells) in complete medium into 96-well u-bottom plates with cell-repellent surface (Greiner Bio-One; Austria). Spheroid cultures were immediately incubated for 48 h at 37°C in an incubator with 5% CO_2_ until spheroid formation.

For phenotyping experiments, positively selected CD14^+^ PBMCs (1 × 10^5^) were antibody stained and analysed by flow cytometry prior to culture with spheroids to determine the baseline (T = 0) myeloid phenotype. CD14^+^ PBMCs (4 × 10^4^) were added to the spheroid cultures on day 2 of spheroid formation and incubated for 48 h alongside M1-like, M2-like, and unpolarised media control Mφs. Spheroid-induced Mφ polarisation was measured by flow cytometry. Viable myeloid cells were gated based on positive CD14 staining and negative Fixable Viability Dye eFluor780 staining (eBioscience; San Diego, CA, United States).

### 2.4. Brightfield Microscopy

After 48 h polarisation, the multi-component spheroid cultures were imaged using a widefield, phase-contrast Time Lapse Axiovert S100 TV microscope (Zeiss; Germany) to study myeloid cell morphology.

### 2.5. Primary Tissue Processing and Explant Culture

Fresh NSCLC tissue and autologous peripheral blood was obtained from patients undergoing surgical resection through the Wales Cancer Bank. Samples were obtained under informed consent and with ethical approval (see above). Samples included in this report were confirmed as NSCLC by a certified histopathologist. Patient demographics corresponding to samples used in this study are shown in Table 1. PBMCs were isolated from fresh peripheral blood (10–20 mL) and CD14^+^ PBMCs isolated by positive selection as described above. Fresh tumour tissues were dissected into ~1mm^3^ explants using a McIlwain Tissue Chopper (Campden Instruments Ltd.; United Kingdom). CD14^+^ PBMCs (1 × 10^5^) were antibody stained and analysed by flow cytometry prior to culture to determine the baseline (T = 0) myeloid phenotype. CD14^+^ PBMCs (1 × 10^5^/well) were co-cultured with 5 explants/well of a 48-well flat-bottom cell-repellent surface plate (Greiner Bio-One; Austria) for 48 h in 1% FBS-containing complete RPMI-1640. M1-like, M2-like, and unpolarised media control Mφs were established in parallel. Explant only wells were setup to assess the in situ TAM phenotype. Explant-induced Mφ polarisation was assessed by flow cytometry. Viable myeloid cells were gated based on positive CD14 staining and negative Fixable Viability Dye eFluor780 staining.

### 2.6. Flow Cytometry

Cells were stained in phosphate-buffered saline (PBS; Sigma-Aldrich; Missouri, United States) with Fixable Viability Dye eFluor780 (1/1000; eBioscience; California, United States) to exclude dead cells potentially contributing to non-specific antibody staining. Staining was performed at 4 °C for 30 min in PBS, in the dark. The non-specific binding of antibodies to Fc receptors was blocked using 2.5% mouse serum (Sigma-Aldrich; Missouri, United States) at room temperature (RT) for 15 min. CD14^+^ PBMCs were then surface-labeled with the following fluorochrome-conjugated, mouse anti-human antibodies: PE/Cy7-CD14 (BioLegend; San Diego, CA, United States), BV421-CD206 (BD Bioscience; California, United States), PerCP/Cy5.5-CD64 (BioLegend; California, United States), and APC-CD163 (eBioscience; California, United States). Staining was performed at 4 °C for 40 min in the dark, in PBS containing 2% FBS and 1 mmol/L EDTA (Sigma-Aldrich; Missouri, United States). Unstained cells were used as a negative control, and fluorescence minus one (FMO) controls were used to identify and remove spectral overlap from quadrant gating analyses. Flow cytometry was performed using an 8-colour FACSVerse flow cytometer with FACSuite software v.1.2.1 (BD Bioscience; California, United States), and the results were analysed using FACSDiva v.6.1.2 (BD Biosciences; California, United States) software. Marker expression is given as the delta mean fluorescence intensity (MFI) for each antibody-labeled sample after subtraction of MFI measured in the corresponding unstained control (values shown in Table S1), and percentage of cells expressing the marker within the live parent population.

### 2.7. T Cell Functional Assay

In order to evaluate the immune modifying effects of tumour spheroid- and explant-conditioned macrophages on T cell function, CD14^−^ PBMCs were cultured with autologous tumour-conditioned Mφs, or control Mφs, from either healthy donors or NSCLC patients. Healthy donor or patient CD14^+^ PBMCs (1 × 10^4^) were co-cultured with heterotypic and homotypic spheroids, or tumour explants (one per well) in adherent 96 well u-bottom plates (Greiner Bio-One, Austria). M1-like, M2-like, and unpolarised media control Mφs were established in parallel. Heterotypic spheroid or explant only (absence of exogenously added CD14^+^ PBMCs) conditions were established in parallel. After 24–48 h of macrophage polarisation treatment, culture-conditioned medium was removed. Mφ cultures were loaded with a pool of viral/recall peptides (and short immunogenic bacterial peptides; tetanus toxoid, TT) (VP; 5 μg/mL) derived from antigens of Influenza A, human Cytomegalovirus (HCMV), and Epstein-Barr virus (EBV) (Severn Biotech Ltd.; Kidderminster, United Kingdom), with a wide variety of HLA (MHC-I and MHC-II) restrictions (epitope sequences shown in Table S2) for 1 h. DMSO (Sigma-Aldrich; Missouri, United States), diluted the same as for the peptides, was added as a control. Cryopreserved autologous CD14^−^ PBMCs were thawed and added (1 × 10^5^ cells/ well) in fresh complete medium to tumour-conditioned cultures. T cells only controls were established in parallel. T cell cultures were supplemented with 20 ng/mL IL-1β (Peprotech; New Jersey, United States) and 1000 U/mL IFNα (R&D Systems; Minnesota, United States), and cultured for 6 days [26,27]. Supernatants were collected, centrifuged (354× *g* for 5 min; Heraeus Megafuge 1.0, Thermo Fisher Scientific, Massachusetts, United States) to remove cell debris, and frozen at −20 °C until required for LEGENDplex^TM^ (BioLegend; California, United States) analysis. T cell cultures were then re-stimulated with 5 μg/mL VP pool in fresh complete medium for 1 h at 37 °C before 1 μL/mL Golgi Plug (BD PharMingen; San Diego, CA, United States) and 0.7 μL/mL Golgi Stop (BD PharMingen; California, United States) were added, followed by 13 h further incubation at 37 °C. Cells were harvested, washed in PBS, and stained with Fixable Viability Dye eFluor780 (eBioscience; California, United States) as described above. Cells were fixed for 15 min at RT in Fixation Buffer (eBioscience; California, United States), washed, and permeabilised using 1× Permeabilisation Buffer (eBioscience; California, United States) containing 2.5% mouse serum (RT; Sigma-Aldrich; St. Louis, MO, United States). After 15 min, cells were stained with PE/Cy7-CD3 (eBioscience; California, United States), BV421-CD4 (BioLegend; California, United States), PerCP/Cy5.5-CD8 (eBioscience; California, United States), FITC-IFNγ (eBioscience; California, United States), and APC-TNFα (eBioscience; California, United States) Staining was performed at RT for 40 min in the dark. Pro-inflammatory cytokine production from T cells was measured by flow cytometry. Viable T cells were gated based on positive CD3 staining and negative Fixable Viability Dye eFluor780 staining.

### 2.8. LEGENDplex^TM^ Bead-Based Immunoassay

Supernatants were thawed on ice and a Human Macrophage/Microglia LEGENDplex^TM^ fluorescence bead-based immunoassay (BioLegend; California, United States) was used to probe and quantify the levels of 13 soluble factors (IL-12p70, IL-12p40, TNFα, IL-6, IL-1β, IL-23, IFNγ, CXCL10, IL-4, IL-10, arginase, CCL17, and IL-1RA) present following T cell co-culture. Raw data were given as the MFI of phycoerythrin (PE) signal. The concentration (pg/mL) of each soluble factor present in culture supernatants was determined by comparing the PE MFI for each target against 13 individual protein standards. Data were analysed using LEGENDplex^TM^ v.8.1 data analysis software (BioLegend; California, United States).

### 2.9. Statistical Analyses

Statistical analyses were performed using Prism v.5.0 software (GraphPad; California, United States), by one-way ANOVA with Tukey post hoc test or two-way ANOVA with Bonferroni multiple comparisons post hoc test. A *p* value < 0.05 was considered statistically significant.

## 3. Results

### 3.1. NSCLC Spheroid-Conditioned Polarisation of Mφs In Vitro: Morphology and Two-Marker Analysis Based on CD64 and CD206 Expression

**Figure 1:**
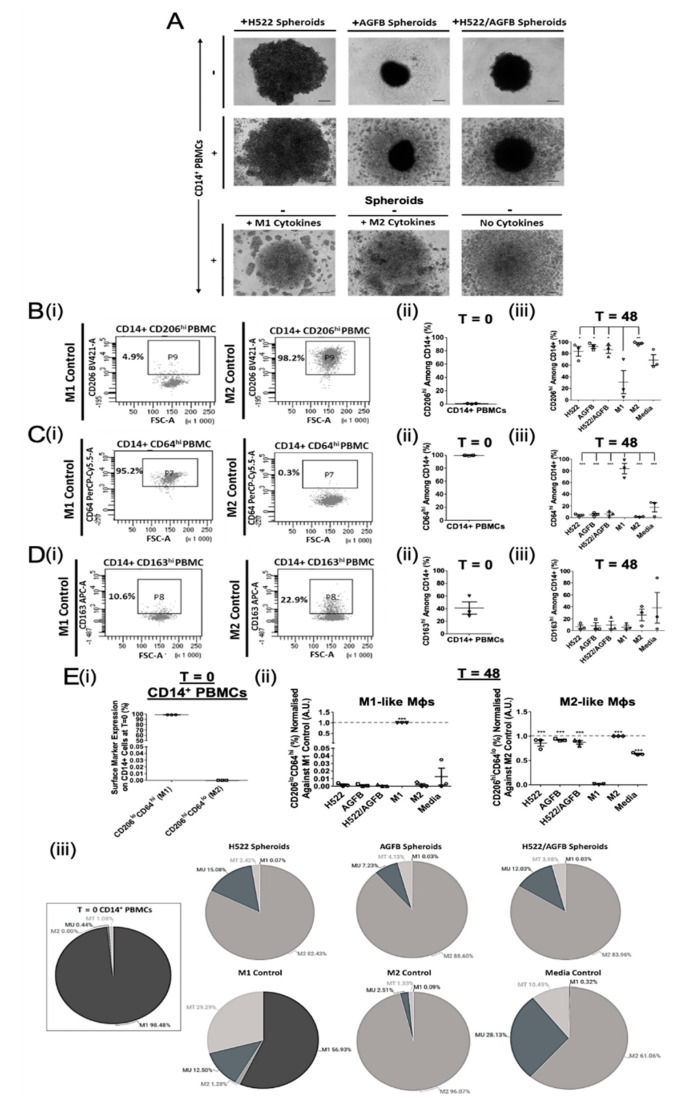
Tumour- and stroma-containing spheroids promote M2-like Mφ polarisation in vitro. H522 cells were cultured alone or together with AG02603 fibroblasts (AGFB) to generate homoTable 48 h formation). Spheroids were co-cultured with healthy donor CD14^+^ PBMCs for a further 48 h to enable macrophage polarisation. CD14^+^ cells were polarised into M1 or M2 Mφ controls, as described above, or incubated without cytokines (unpolarised media controls). (**A**) Morphological assessment of the single- or multi-component spheroid cultures after 48 h polarisation (representative of three independent experiments). Scale bars represents 150 µm. (**B**–**D**) (**i**) Flow cytometry analysis of CD206, CD64, and CD163 expression on CD14^+^ PBMCs following 48 h polarisation. Summary of (**B**) CD206, (**C**) CD64, and (**D**) CD163 expression on myeloid cells (**ii**) at T = 0 and (**iii**) following 48 h spheroid co-culture (matched to T = 0) or cytokine treatment. (**E**) Summary of the proportion of M1- or M2-like myeloid cells (**i**) at T = 0 and (**ii**) 48 h after spheroid co-culture or cytokine treatment (matched to T = 0), determined using two-marker analyses normalised against the respective positive control. **E** (**iii**) Pie charts representing the proportion of CD14^+^ cells exhibiting an M1-, M2-, MU-, and MT-like phenotype following 48 h in vitro culture for all experimental conditions. MT, Transitional myeloid cells; MU, Unpolarised myeloid cells. Each symbol represents an individual patient. The *p* values were obtained from one-way ANOVA followed by post hoc Tukey test. Statistically significant differences were observed compared to the M2 control ((**E**) (**ii**); M1-like Mφs), M1 control ((**E**) (**ii**); M2-like Mφs), or all experimental arms (**B**–**D** (**iii**)). Data were obtained from 3 independent experiments using healthy donor blood and graphs show means ± SEM. * *p* < 0.05, ** *p* < 0.01, *** *p* < 0.001.

To investigate the effect of NSCLC and supporting fibroblast spheroids on Mφ polarisation, healthy donor CD14^+^ PBMCs were cultured either with homotypic or heterotypic tumour- and stroma- containing spheroids (H522; AGFB; H522/AGFB) for 48 h. The morphology of the different spheroids was studied using brightfield microscopy (Figure 1A). H522 cells generated less compact spheroids than AGFB cells. The formation of more compact spheroids upon the addition of stromal cell to tumour spheroids has been observed previously [16]. The addition of CD14^+^ PBMCs to homotypic H522 spheroids resulted in spheroid expansion, whilst their addition to homotypic AGFB or heterotypic H522/AGFB spheroids did not affect spheroid size (Figure 1A, first vs. second row). CD14^+^ PBMC cultures could also be distinguished morphologically, based on myeloid cell clustering, depending on the exogenous cytokine treatment they had received (Figure 1A, third row). Specifically, M1-like Mφs (IFNγ/LPS induced) formed fewer, larger clusters than M2-like Mφs (IL-4/IL-13/IL-10 induced). In contrast, the unpolarised media control Mφs showed diffused or no clustering. Such observations may be a result of differences in cell-cell interaction features between M1- and M2-like Mφ cultures.

Myeloid cells were harvested following 48 h co-culture and phenotyped by flow cytometry (Figure 1B–D). First, we assessed whether M1- and M2-like subtypes can be distinguished by differential expression of CD206, CD63, or CD163 markers.

The M2 marker, CD206, was expressed at almost negligible levels (0.8 ± 0.3%; MFI 289.4 ± 115.1) at baseline (T = 0; Figure 1B(ii)). CD206 was upregulated on all myeloid cells following 48 h culture either with spheroids, M2 polarising cytokines (97.1 ± 1.5%; MFI 24820.0 ± 10777.0), or complete medium with no cytokines (68.5 ± 9.3%; MFI 3384.3 ± 1593.7) (Figure 1B(iii)). M2 Mφs expressed the highest levels of CD206, which were about 3-fold higher than that on M1 Mφs (30.8 ± 19.8%; MFI 1545.1 ± 1144.6). All spheroid-conditioned Mφs expressed significantly more CD206 than the M1 control (H522 83.6 ± 8.3%, MFI 5777.8 ± 2287.3; AGFB 92.0 ± 3.3%, MFI 10875.0 ± 4186.5; H522/AGFB 86.9 ± 6.6%, MFI 5582.6 ± 2008.9). The results show that myeloid cells preferentially polarise into an M2-like Mφ phenotype following exposure to tumour- or stroma-containing spheroids.

In contrast, myeloid cells at baseline (T = 0) expressed high levels of the M1 marker CD64 (99.3% ± 0.2; MFI 7770.5 ± 4067.5; Figure 1C(ii)), which were reduced in each experimental arm upon culture (1.6–17.6%; MFI 233.5–520.1; Figure 1C(iii)). CD64 expression remained >4-fold higher on M1 Mφ controls (83.6 ± 8.9%; MFI 4782.8 ± 645.4) than on unpolarised media control Mφs (17.6 ± 7.8%; MFI 520.1 ± 165.3). CD64 downregulation confirms the M2-like Mφ skewing effect of tumour- and stroma-containing spheroids.

Interestingly, the routinely described M2 marker, CD163 appeared to be moderately expressed on T = 0 healthy donor myeloid cells (41.0 ± 9.7%; MFI 348.6 ± 385.6; Figure 1D(ii)) but was downregulated on spheroid-, cytokine-, and media-conditioned myeloid cells following 48 h culture. There was no significant downregulation of CD163 on unpolarised Mφs (38.3% ± 25.8; MFI 1785.5 ± 1118.7) (Figure 1D(iii)). Importantly, whilst M2-like Mφs expressed higher levels of CD163 (26.3% ± 9.7, MFI 1799.8 ± 1139.9) than the M1 controls (5.7 ± 3.1%; MFI 101.7 ± 133.4) or spheroid-conditioned arms (Figure 1D(iii)), the differences were not significant. These results demonstrate that tumour-induced Mφ polarisation cannot be reliably assessed using CD163 as a single M2 marker.

In an attempt to better define the TAM phenotype, two-marker flow cytometry analyses of CD206 and CD64 expression were performed on all groups (representative dot plots shown in Figure S1A). CD206^lo^CD64^hi^ cells were considered M1-like, while CD206^hi^CD64^lo^ cells represented M2-like Mφs. Unpolarised (MU; CD206^lo^CD64^lo^) and transitional (MT; CD206^hi^CD64^hi^) myeloid cell populations were also assessed based on the quadrants of the two-marker analyses.

The majority of circulating CD14^+^ cells appeared M1-like (98.5%) at baseline, with no evidence for the presence of M2-like cells (0.0%) (Figure 1E(i)). A small proportion of T = 0 myeloid cells exhibited intermediate MU- (0.4%) and MT-like (1.1%) phenotypes (Figure 1E(iii)). Spheroid-conditioned Mφs were predominantly M2-like (82.4–88.6%), however a minor proportion of MU- (7.2–15.1%) and MT-like (2.4–4.1%) cells were also observed in these cultures (Figure 1E(ii,iii). M1-like cells were not detectable in spheroid-conditioned Mφ cultures (0.0–0.1%).

M1-polarised Mφs possessed a higher degree of plasticity than the M2 polarised Mφs, as 29.3% of cells in this treatment group displayed an MT-like phenotype, expressing high levels of both CD206 and CD64 following culture, compared to 1.3% in the M2 control group (Figure 1E(iii)). Interestingly, whilst 28.1% of media control CD14^+^ PBMCs exhibited an MU-like phenotype, the majority of cells were polarised into M2-like Mφs (61.1%) (Figure 1E(iii)), albeit with a low CD206 expression (Media and M2 control MFI: 3384.3 ± 1593.7 and 24820.0 ± 10777.0, respectively). Overall, these results indicate that two-marker flow cytometry analyses, using the combination of CD206 and CD64, but not CD163, can better reveal the nature and plasticity of the TAM phenotype than single markers.

### 3.2. Ex Vivo Study of Mφ Polarisation Induced by Lung Tumour Explants, Using the Two-Marker Approach

**Figure 2:**
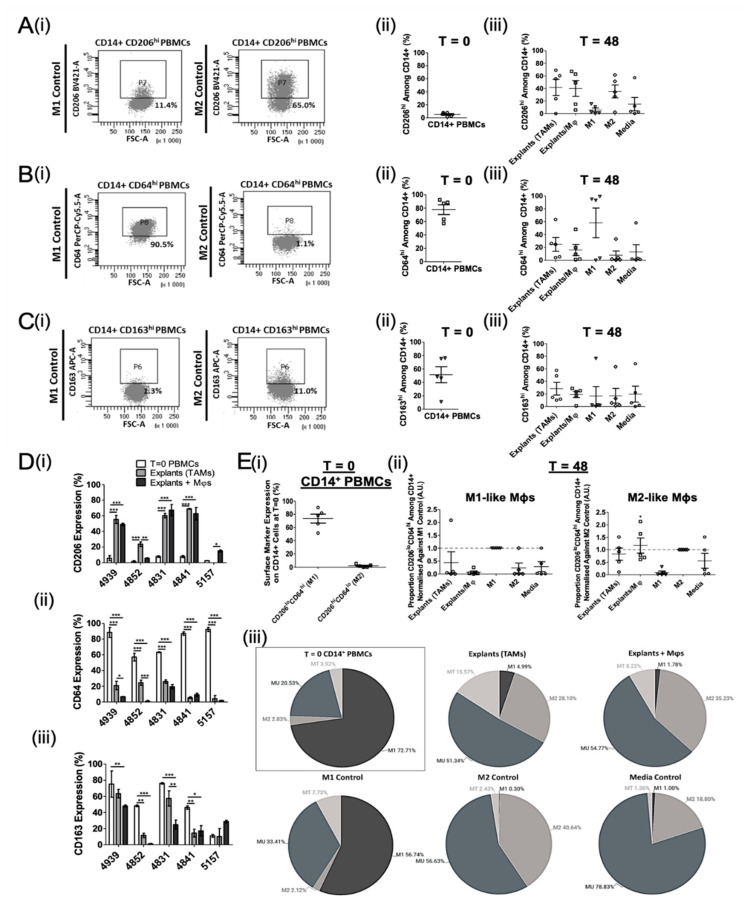
NSCLC explants promote M1- to M2-like Mφ polarisation ex vivo. Tumour explants were co-cultured with autologous patient CD14^+^ PBMCs for 48 h to enable macrophage polarisation. CD14^+^ cells were polarised into M1 or M2 Mφ controls, as described, or incubated without cytokines (unpolarised media controls). Explant only wells were setup to assess the tumour-associated macrophage (TAM) phenotype. The M1- and M2-like Mφ phenotype before (T = 0) and after 48 h culture (T = 48) was analysed by flow cytometry. (**A**–**C**) (**i**) Representative flow cytometry plots of CD206, CD64, and CD163 expression on CD14^+^ PBMCs at T = 48. Summary of (**A**) CD206, (**B**) CD64, and (**C**) CD163 expression on myeloid cells (**ii**) at T = 0 and (**iii**) following 48 h culture with tumour explants or cytokines (matched to T = 0). (**D**) Combined T = 0 and T = 48 expression data for each patient. Changes in (**i**) CD206, (**ii**) CD64, and (**iii**) CD163 levels on CD14^+^ PBMCs following exposure to explants are shown. (**E**) Summary of the proportion of M1- or M2-like myeloid cells (**i**) at T = 0 and (**ii**) generated by explant co-culture (matched to T = 0), determined using two-marker analyses normalised against the respective positive control. Each symbol represents an individual patient. (**E**) (**iii**) Pie charts representing the proportion of CD14^+^ cells exhibiting an M1-, M2-, MU-, and MT-like phenotype following 48 h ex vivo culture for all experimental conditions. MT, Transitional myeloid cells; MU, Unpolarised myeloid cells. The *p* values were obtained from one-way ANOVA followed by post hoc Tukey test (**A**–**C**,**E**) and two-way ANOVA followed by Bonferroni post hoc test (**D**). Statistically significant differences were observed compared to the M2 control (**E**) (**ii**); M1-like Mφs), M1 control (**E**) (**ii**); M2-like Mφs), or all experimental arms (**A**–**D**). Data were obtained from 5 independent experiments using patient blood and tissue and graphs show means ± SEM. * *p* < 0.05, ** *p* < 0.01, *** *p* < 0.001.

Two-component tumour spheroid models, using cell lines, do not fully recapitulate the cellular heterogeneity and complexity of immune interactions within the TME. Therefore, to establish the Mφ-polarising potential of the lung TME, we developed an ex vivo NSCLC explant model using fresh tumour tissue. As such, we assessed both the tissue-resident TAM profile and the Mφ phenotype induced by polarisation of exogenously added, autologous myeloid cells in a 48 h explant culture. Mφs were harvested from the explant cultures and phenotyped by flow cytometry (Figure 2). As seen previously with healthy donor CD14^+^ PBMCs (Figure 1B(ii)), baseline (T = 0) CD206 expression was low (5.2 ± 1.7%; MFI 119.7 ± 147.4; Figure 2A(ii)) and appeared to be upregulated on myeloid cells following 48 h culture in all experimental arms (5.9–41.64%; MFI 267.2–1130.5). Both tissue resident TAMs (explants without exogenous addition of CD14^+^ cells) and recovered explant-conditioned CD14^+^ myeloid cells expressed CD206 levels similar to the M2 controls. M1 and media control Mφs showed low levels of CD206 expression, indicating that both TAMs and explant-conditioned Mφs tend to be M2-like in NSCLC.

CD64 was highly expressed on myeloid cells at T = 0 (77.6 ± 8.9%; MFI 1170.2 ± 427.2) and was downregulated on cells following 48 h culture with explants, M2 cytokines, or complete medium (7.9–15.9%; MFI −878.7–362.3) (Figure 2B), being highest on M1 controls and lowest on M2 controls. TAMs and explant-conditioned Mφs appeared more M2-like than M1-like based on mean CD64 expression (Figure 2B(iii)).

The classic M2 marker, CD163 was moderately expressed on myeloid cells at baseline (51.4 ± 18.8%; MFI 1170.2 ± 427.2; Figure 2C(ii)), but was downregulated following 48 h culture with explants, polarising cytokines, or medium (16.6–19.7%; MFI −349.8–356.0) (Figure 2C(iii)). Although TAMs had the highest levels of CD163 (28.5 ± 15.1%; MFI 1175.5 ± 709.2), the mean CD163 expression (MFI and percentage CD163^hi^ cells) was comparable for all treatment conditions.

When taking the T = 0 and 48 h polarisation data for each of the 5 patient explant experiments individually, CD206 was highly expressed on TAMs and explant-conditioned Mφs compared to T = 0 cells in 4/5 and 5/5 of donors, respectively (Figure 2D(i)). In contrast, both CD64 and CD163 expression was downregulated on TAMs and explant-conditioned Mφs compared to T = 0 myeloid cells in 4/5 of donors (Figure 2D(ii,iii)). Overall, this demonstrates that TAMs exhibit an overwhelmingly M2-like phenotype in the lung TME, and tumour explants promote M2-like Mφ polarisation ex vivo. Additionally, changes in CD163 expression are not informative to determine an M2-shift in either the spheroid or explant model.

In order to better discriminate between the M1- and M2-like Mφ phenotype, two-marker flow cytometry analysis of CD206 and CD64 was performed to identify transitional (MT) or unpolarised myeloid cell (MU) populations as well (representative dot plots shown in Figure S1B).

The majority of circulating CD14^+^ cells appeared M1-like (CD206^lo^CD64^hi^; 72.7%) at baseline, with minimal evidence for the presence of M2-like cells (CD206^hi^CD64^lo^; 2.8%) (Figure 2E(i)). Interestingly, 20.5% and 3.9% of these T = 0 myeloid cells exhibited an MU- (CD206^lo^CD64^lo^) and MT-like (CD206^hi^CD64^hi^) phenotype, respectively (Figure 2E(iii)). In contrast, TAMs (i.e., the TME) were composed of more M2- (28.1%) than M1-like (5.0%) Mφs (Figure 2E(ii)). However, the majority of TAMs exhibited an MU-like phenotype (51.3%), although an additional 15.6% appeared MT-like after 48 h culture (Figure 2E(iii)). This was partially reflected in the “Explants+ Mφs” group, which included 1.8% M1-like, 35.2% M2-like, 8.2% MU-like, and 54.8% MT-like cells (Figure 2E(ii,iii)), indicating complex environmental effects of the TME on Mφ polarisation.

### 3.3. Mφ Plasticity in the TME Model

**Figure 3:**
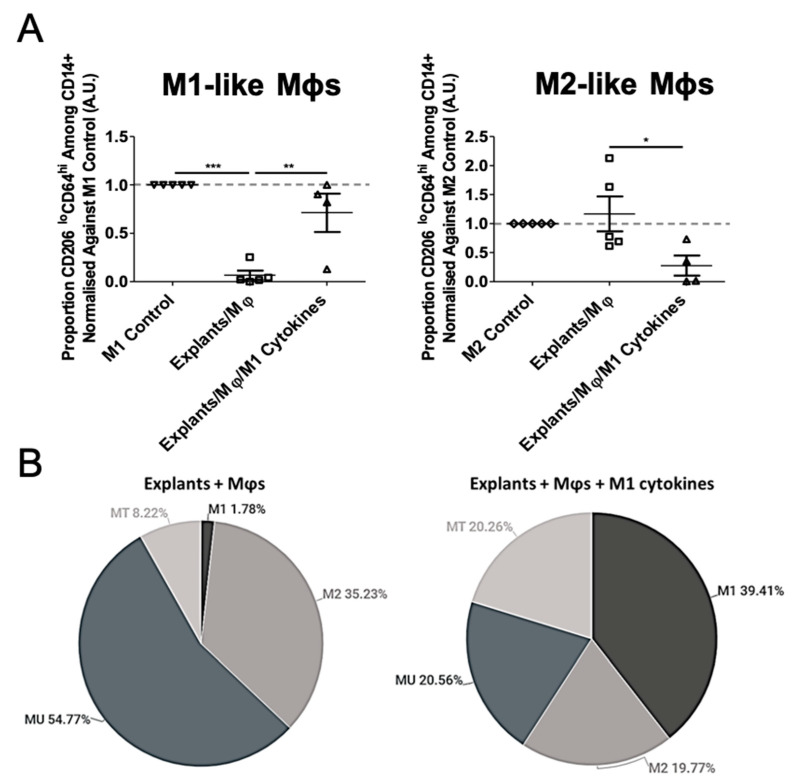
Explant-induced M2-like Mφ polarisation is reversed upon LPS and IFNγ treatment. (**A**) To test the plasticity of the explant-induced M2-like phenotype, explant-conditioned Mφ co-cultures were treated in parallel with or without M1-promoting cytokines (20 ng/mL IFNγ and 10 pg/mL LPS) for 48 h. Each symbol represents an individual patient. (**B**) Pie charts representing the proportion of CD14^+^ cells exhibiting an M1-, M2-, MU-, and MT-like phenotype following 48 h ex vivo culture with explants in the absence (**left**) and presence (**right**) of M1-promoting cytokines. MT, Transitional myeloid cells; MU, Unpolarised myeloid cells. The *p* values were obtained from one-way ANOVA followed by post hoc Tukey test. Statistically significant differences were observed compared to the M2 control (M1-like Mφs) or M1 control (M2-like Mφs). Data were obtained from 5 independent experiments using patient blood and tissue and graphs show means ± SEM. * *p* < 0.05, ** *p* < 0.01, *** *p* < 0.001.

In order to determine the stability of the arising M2 phenotype, and the plasticity of the M1/M2 state, we examined whether explant-induced M2-like Mφs could be repolarised into an M1-like phenotype through treatment with M1-promoting cytokines (IFNγ and LPS). In explant-conditioned Mφ cultures, M1-polarising cytokine treatment significantly increased the proportion of M1-like Mφs (CD206^lo^CD64^hi^; 1.8% vs. 39.4% before and after treatment, respectively), and decreased M2-like Mφs (CD206^hi^CD64^lo^; 35.2% vs. 19.8% before and after treatment, respectively) (Figure 3A). The treatment additionally promoted an increase in MT-like cells (CD206^hi^CD64^hi^; 8.2% vs. 20.3% before and after treatment, respectively), and a decrease in MU-like cells (CD206^lo^CD64^lo^; 54.8% vs. 20.6% before and after treatment, respectively), present in explant-conditioned Mφ cultures, however the latter did not reach statistical significance (Figure 3B). Overall, these results show the plasticity of the M2 phenotype and the ability to manipulate the system to achieve a desirable M1-like subtype within the TME. Therefore, the NSCLC explant model has the potential for use in immunomodulation studies ex vivo.

### 3.4. Tumour Explant, but Not Spheroid, Co-Cultures Suppress CD8^+^ T Cell Responses

**Figure 4:**
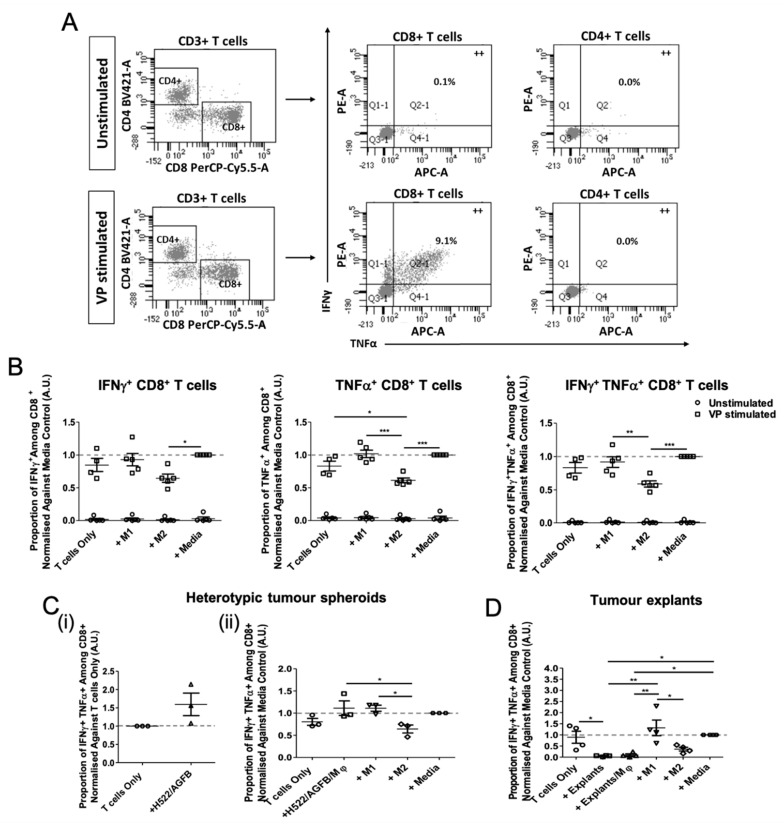
Tumour explants, but not spheroids suppress CD8^+^ T cell activity. CD14^−^ PBMCs were cultured with autologous tumour-conditioned Mφs, or control M1/M2/Media Mφs, from either healthy donors or NSCLC patients. Mφ cultures were loaded with 5 μg/mL viral peptide pool (VP stimulated) or DMSO (unstimulated) prior to the addition of T cells. T cell co-cultures were incubated for 6 days. Intracellular cytokine staining of IFNγ and TNFα was performed and analysed by flow cytometry. (**A**) Representative gating strategy of CD3^+^ cells for flow cytometry analysis of pro-inflammatory cytokine production (++ = double positive staining) by CD4^+^ and CD8^+^ T cells. Summary of the proportion of IFNγ^+^, TNFα^+^, and IFNγ^+^TNFα^+^ cytokine production from CD8^+^ T cells in the presence or absence of (**B**) M1, M2, and unpolarised media control Mφs (*n* = 5 healthy donors), (**C**) Heterotypic tumour spheroids (in the (**i**) absence and (**ii**) presence of exogenously added Mφs; *n* = 3 healthy donors), and (**D**) tumour explants/explant-conditioned Mφs (*n* = 4 NSCLC patients). Each symbol represents an individual healthy donor or patient. Values normalised against unpolarised media control Mφs. The *p* values were obtained from one-way ANOVA followed by post hoc Tukey test. Statistically significant differences were observed compared to all experimental arms. All data were pooled from at least 3 independent experiments and graphs show means ± SEM. * *p* < 0.05, ** *p* < 0.01, *** *p* < 0.001.

In order to assess the effects of spheroid- and explant-conditioned Mφs on T cell activity, tumour-conditioned or control Mφs were loaded with viral peptides (VP) and co-cultured with CD14^−^ PBMCs for 6 days. This approach has been shown to stimulate existing T cell memory in an antigen-specific manner (26). Pro-inflammatory cytokine production (IFNγ and TNFα) from CD8^+^ or CD4^+^ T cells was assessed by flow cytometry (Figure 4A). The VP pool (consisting mainly of 9-mer peptides) dominantly stimulated IFNγ and TNFα production from CD8^+^ T cells and not CD4^+^ T cells. No cytokine production was observed from unstimulated T cell cultures either (Figure 4B). In general, the proportion of CD8^+^ T cells producing pro-inflammatory cytokines was significantly lower in the presence of M2-like than M1-like Mφs, while the proportion of IFNγ^+^, TNFα^+^, and IFNγ^+^TNFα^+^ CD8^+^ T cells was comparable when cultured with M1 control Mφs, unpolarised Mφs (media controls), or complete medium (T cells only). The results demonstrate that M2-like Mφs suppress CD8^+^ T cell responses.

Heterotypic spheroids alone did not suppress CD8^+^ T cell function in vitro (Figure 4C(i)). Despite spheroid-conditioned Mφs appearing M2-like in phenotype, these Mφ co-cultures did not affect T cell stimulation compared to that by M1-like Mφs (Figure 4C(ii)).

In contrast, tumour explants, both in the presence or absence of exogenously added autologous myeloid cells, supported significantly lower T cell stimulation, compared to that by M1 and media Mφ controls (Figure 4D). This demonstrates that the suppressive nature of the TME is dominant and could not simply be reversed by the addition of exogenous Mφs. Whilst the data shown here suggest that exogenously added myeloid cells do not actively promote a pro-inflammatory lung TME, the additional contribution of Mφs to localised immunosuppression could not be concluded using the T cell suppression assay due to the dominant immunoinhibition of explants alone.

**Figure 5:**
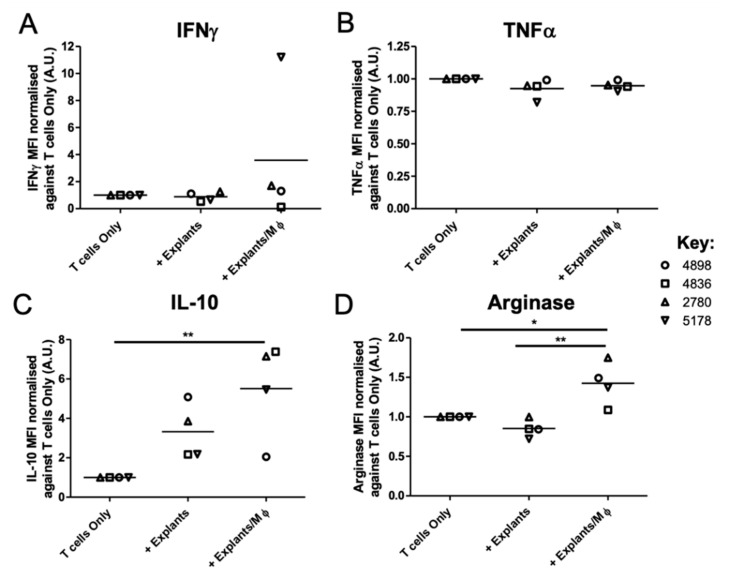
Tumour-conditioned Mφs contribute to the development of an immunosuppressive lung tumour microenvironment ex vivo. NSCLC explants were co-cultured with autologous patient CD14^+^ PBMCs for 48 h to enable macrophage polarisation. Explant only wells were established to assess soluble factor secretion from tumour-associated Mφs and other tumour microenvironment components. Tumour cultures were loaded with 5 μg/mL viral peptide pool prior to the addition of T cells. T cell co-cultures were incubated for 6 days. Supernatants were harvested from each culture condition and a Human Macrophage/Microglia LEGENDplex^TM^ bead-based immunoassay was used to quantify the levels of 13 soluble factor targets released from the T cell co-culture system. The mean fluorescence intensity (MFI, A.U.) values for (**A**) IFNγ, (**B**) TNFα, (**C**) IL-10, and (**D**) arginase, are shown. Values normalised against T cell Only outputs. Each symbol represents an individual patient (*n* = 4 NSCLC patients). The *p* values were obtained from one-way ANOVA followed by post hoc Tukey test. Statistically significant differences were observed compared to all experimental arms. Data are pooled from 4 independent patient experiments and graphs show means ± SEM. * *p* < 0.05, ** *p* < 0.01.

To further explore the additive suppressive capacity of explant-conditioned Mφs to localised immunosuppression, the secretion of 13 myeloid cell-related Th1 and Th2 soluble factors by explant-conditioned T cell co-cultures were measured in the presence and absence of exogenously added patient Mφs. The MFI (Figure 5 and Table S3A) and concentration (Table 2 and Table S3B) of IL-12p70, IL-12p40, TNFα, IL-6, IL-1β, IL-23, IFNγ, CXCL10, IL-4, IL-10, arginase, CCL17, and IL-1RA in culture supernatants was determined via LEGENDplex^TM^ analysis.

The secretion of pro-inflammatory cytokines from explant-conditioned T cell co-cultures remained unchanged upon the addition of exogenous Mφs to the ex vivo system (Table S3), including the levels of IFNγ (+ Explants: 3126.6 ± 1747.3 MFI; + Explants/Mφ: 3733.1 ± 2123.0 MFI) and TNFα(+ Explants: 173.1 ± 4.0 MFI; + Explants/Mφ: 177.6 ± 4.9 MFI) (Figure 5A,B). The concentration of IFNγ detected in explant-conditioned T cell culture supernatants ranged from 0.6–150.7 pg/mL and 1.1–209.3 pg/mL in the absence and presence of exogenously added myeloid cells, respectively (Table 2). Similarly, TNFα concentrations ranged from <1.2–2.0 pg/mL and <1.2–1.6 pg/mL in the absence and presence of exogenously added myeloid cells, respectively (Table 2). These data are in keeping with the T cells suppression assay data, shown in Figure 4D.

In contrast, the levels of the anti-inflammatory molecules, IL-10 (+ Explants: 495.4 ± 37.4 MFI; + Explants/Mφ: 1036.2 ± 337.3 MFI) and arginase (+ Explants: 32306.1 ± 2235.2 MFI; + Explants/Mφ: 53625.3 ± 3946.0 MFI) produced from T cell cultures was significantly increased in the presence of explant-conditioned Mφs, but not explants alone (Figure 5C,D). The concentration of IL-10 detected in explant-conditioned T cell culture supernatants ranged from 2.7–4.0 pg/mL and 1.7–17.0 pg/mL in the absence and presence of exogenously added myeloid cells, respectively (Table 2). Similarly, arginase concentrations ranged from 2346.5–3286.0 pg/mL and 4213.0–5712.6 pg/mL in the absence and presence of exogenously added myeloid cells, respectively (Table 2). No other Th2-related factors were notably changed following the addition of exogenous Mφs to explant-conditioned T cell co-cultures (Table S3).

Overall, our findings indicate an additive contribution of Mφs to the development of an immunosuppressive lung TME ex vivo by increasing the ratio of Th2: Th1 factors present in culture. Whilst both the NSCLC spheroid and explant model appeared to demonstrate similar tumour-induced M2 skewing of Mφ polarisation, the functional data indicate that heterotypic tumour spheroid-conditioned Mφ co-cultures are an oversimplified model of the lung TME since they do not affect T cell stimulation. In contrast, the explant approach induces currently unclassified changes during Mφ polarisation that are associated with regulation of T cell activity. Here, we conclude that the tumour explant model better represents the complex interplay of tumour-immune interactions which occur within the lung TME, compared to the heterotypic spheroid model. The data from this study demonstrate the potential utility of our model in further studying the specific contribution of cellular components to T cell suppression in the TME, ex vivo.

## 4. Discussion

A variety of myeloid cell subsets are known to mediate immune suppression, tumour invasion, and neovascularisation within the TME, including: TAMs, tumour-associated neutrophils, tumour-associated dendritic cells, Tie2-expressing monocytes, and myeloid-derived suppressor cells (MDSCs) [28,29]. The focus of this study was restricted to TAMs since these are the most abundant tumour-infiltrating myeloid cells in NSCLC tissue, constituting up to 50% of tumour mass [15,30].

M2-like Mφs have been shown to promote an immunosuppressive TME which supports tumourigenicity and angiogenesis in in vitro and in vivo models of NSCLC [6,8]. The work presented here supports these findings, describing a dynamic interplay between the tumour, stroma, and immune cells in a 3D spheroid and novel ex vivo tumour explant setting, resulting in the promotion of an M2-like Mφ phenotype [15]. Our data support the use of the tumour explant model in future immunomodulation studies since the heterotypic spheroid model may generate misleading data as it does not fully encapsulate the dynamic interactions between various immune compartments and the tumour in the lung TME.

CD14^+^ PBMCs incubated with fibroblast-containing spheroids appeared more M2-like than those incubated with homotypic tumour spheroids. This suggests an important function for stromal cells in modulating Mφ phenotypes and functions. Fibroblasts may secrete chemokines such as macrophage colony-stimulating factor (M-CSF) which in turn can stimulate alternatively activated Mφs to produce growth factors including transforming growth factor β1 (TGFβ1) [31,32] and platelet-derived growth factor (PDGF) to reciprocally stimulate fibroblasts further [31]. Tumour cells do not act in isolation to exert pro-tumour effects within the TME but rather, are highly dependent on cancer-associated stroma to elicit TAM skewing [3] and myeloid-mediated immunosuppression [33]. Interestingly, even in the absence of tumour, the homotypic stroma-containing spheroids promoted the greatest M2-like skewing effect. Unlike normal fibroblasts, cancer-associated fibroblasts (CAFs) are characterised by their myofibroblast-like phenotype. Stimulation by soluble [34,35] or cancer-derived exosome-tethered [36,37] TGFβ1, or increased mechanical tension in the tissue [34,35], results in fibroblast to myofibroblast differentiation. Therefore, the increased mechanical tension and adhesion structures that form between cells upon spheroid formation may strongly influence the M2-promoting cytokine profile produced by the spheroid model. Hence, generating a synthetic mimic of the TME using a spheroid approach is very challenging, and is likely only to be partly successful given these complexities.

In the literature, there appears to be limited consistency in the markers used to define M1- and M2-like Mφ subsets. Large panels of phenotyping markers have been developed and reported, but many of these only convolute the system further, since a variety of published markers are often expressed on both M1 and M2 Mφs under different conditions. Temporal plasticity in Mφ activation states exist; in particular, arginase-1, a common marker of alternatively activated (M2) Mφs, can also be a late marker of classically activated (M1) Mφs if measured 24h post-stimulation [38]. If measured too early, such markers would not be expressed at all, potentially explaining our CD163 expression findings. Additionally, it has been argued that distinct M1/M2 Mφ populations do not exist in vivo, but instead, they are artifacts of the extreme M1- and M2-promoting conditions created in vitro [39,40]. Due to the remarkable plasticity of the Mφ phenotype, it is believed that at any given time there will be a variety of different Mφs expressing different M1- and M2-type markers [40], so within the TME it is believed that TAMs may exist as intermediate phenotypes, in terms of marker or cytokine expression [41]. Indeed, we observed TAMs with M2 (73%), MT (17%), and MU (9%) phenotypes.

CD163 is a member of the scavenger receptor cysteine-rich (SRCR) family and is exclusively expressed on cells of the myeloid lineage. As such, CD163 is commonly reported to be expressed on non-classical CD14^+^CD16^+^ monocytes, as well as on anti-inflammatory M2-like Mφs [42]. Previous studies have claimed that spheroids containing both NSCLC cells and CAFs skewed myeloid cells into an M2-like TAM state based on CD163 and CD206 expression. However, less than 50% of cells were reported as CD163^+^ following exposure to the multi-component spheroid system [15], indicating that CD163 is not a specific marker of M2-like Mφs. To date, limited studies use M1 or M2 positive controls when concluding the M2 skewing effects of the TME based on CD163 expression and therefore, the specificity and usefulness of CD163 as an M2 Mφ marker is uncertain [3,42]. Despite this, CD163 is more commonly used than CD206 to examine M2-like TAMs within clinical NSCLC tissue sections. In particular, the TAM phenotype is often determined using CD68 (macrophage marker), iNOS (M1) and CD163 (M2) antibodies, respectively [43,44]. It is possible that the CD163 antibodies are better optimised for immunohistochemistry (IHC) than flow cytometry, and that the processing steps of IHC enables the antigen to be better exposed and detected. There have been wide discrepancies in the reported levels of surface CD163 expression in blood myeloid cells using flow cytometry. Indeed, levels of monocytic CD163 expression were observed to be higher when using monoclonal flow cytometry antibodies that recognised epitopes in the N-terminal part of the receptor, and antibody binding affinities differed depending on the extracellular calcium levels when staining [45]. Evidently, the most optimal methodology for assessing particular M1- and M2-like Mφ markers needs to be deciphered and explored in future studies to improve the standardisation of TAM phenotyping.

It was evident from our initial expression data that the analyses of single markers alone is unlikely to be sufficient to classify M1- and M2-like Mφs. A limited number of studies have used a combinational marker approach to better distinguish between un-committed, M1, and M2 Mφ subsets. Previously reports identified Th1-stimulated, M1-like Mφs to be predominantly CD64^+^CD80^+^ (~60–70% depending on Th1 stimulus) and Th2-stimulated, M2-like Mφs to be CD11b^+^CD209^+^ (~65–70% depending on Th2 stimulus) [46]. Additionally, 50% of CD11b^+^CD209^+^ cells expressed CD200R, a marker often associated with the M2 phenotype [46,47], indicating that multi-marker analyses may be required to sufficiently identify an M2 dominant Mφ population. This, together with the findings of this study, strengthen the argument that categorising cells into M1- and M2-like categories may be oversimplifying the plasticity of the Mφ phenotype.

Due to the uncertainty surrounding the use of phenotyping markers to distinguish M1/M2 subsets, our data demonstrate that determining the pro- or anti-inflammatory function of tumour-conditioned Mφs or TAMs is an important method of confidently concluding the presence and role of particular macrophage subsets within the TME. However, only a limited number of research groups have attempted Mφ and T cell functional assays in a human system, making interpretation of the impact of their results more challenging. Whilst some human macrophage studies have successfully re-polarised TAMs into an M1-like state phenotypically [15,48], the effect of these immune-modifying agents on releasing TAM-induced immunosuppression within the TME has yet to be fully explored.

Whilst TAM-mediated immunosuppression has been strongly associated with anti-inflammatory and pro-tumour activity, both macrophage-dependent and macrophage-independent suppression of T cell functions exist within the TME [49]. Within the TME, a high abundance of regulatory T cells, MDSCs, or CAFs, or presence of T cell exhaustion as a result of their chronic engagement with cancer antigens, also contribute to the promotion of an immunosuppressive state [3,49,50]. The results of this study demonstrate that our explant model is capable of recapitulating both tumour-mediated and tumour-conditioned Mφ-mediated inhibition of effective CD8^+^ T cell responses, ex vivo. Such dominant immunosuppression was not observed when using the spheroid model. Whether T cell inhibition is a result of active immunosuppression, mediated by multiple elements of the tumour explant, or due to sub-optimal T cell stimulation needs to be elucidated in future studies.

Interestingly, Mφs polarised by heterotypic pancreatic tumour spheroids were previously observed to suppress CD4^+^ and CD8^+^ T cell proliferation in vitro [16]. However, T cell function, rather than T cell fitness, has not been extensively studied in the presence of tumour-conditioned Mφs generated in an in vitro 3D cancer system.

In conclusion, we developed and characterised multi-component spheroid and explant models to study Mφ polarisation in NSCLC. We demonstrated that CD206 and CD64, used in combination, can reliably distinguish between M1 and M2 Mφ populations, indicating the importance of multi-marker analyses when assessing Mφ phenotypes. Through the establishment and use of a novel two-marker phenotypic macrophage analysis approach, we confirmed that the lung TME dominantly skews CD14^+^ myeloid cells into an M2-like TAM state both in vitro and ex vivo. Our data support the use of the NSCLC explant model over the heterotypic spheroid model, since the former can also demonstrate the immunosuppressive nature of the TME, and advocate an explant approach for future studies of TAM phenotype and function. These findings provide a rationale for targeting M2-like Mφ polarisation in the explant model using immune-modifying agents, in an attempt to reduce potent macrophage-induced immunosuppression within the TME and improve biological response to conventional and immunotherapeutic agents.

## Figures and Tables

**Table 1:** Demographics of patients corresponding to samples included in explant experiments.

Demographic	All Patients *n* = 11
Age at operation, years	
Median (Min, Max)	72.0 (58.0, 78.0)
Mean	71.3
SD	6.3
Sex	
Male	6 (54.5%)
Female	5 (45.5%)
Histology	
Adenocarcinoma	3 (27.3%)
Squamous cell carcinoma	8 (72.7%)
T stage	
T2a	4 (36.4%)
T2b	2 (18.1%)
T3	5 (45.5%)
N stage	
N0	6 (54.5%)
N1	4 (36.4%)
N2	1 (9.1%)
M stage	
MX	7 (63.6%)
M0	3 (27.3%)
M1b	1 (9.1%)

**Table 2:** The mean concentration (pg/mL) of each target present in culture supernatants was determined using individual protein standards for each of the 4 patient (4898, 4836, 2780, and 5178) experiments. Secretion of all other factors remained unchanged (Table S3).

Cytokine/Chemokine Concentration (pg/mL)				
**IFNγ**				
	**4898**	**4836**	**2780**	**5178**
**T cells Only**	<0.53	212.47	113.23	3.32
**+ Explants**	0.62	105.40	150.66	1.55
**+ Explants/Mφ**	1.13	18.65	209.30	78.56
**TNFα**				
	**4898**	**4836**	**2780**	**5178**
**T cells Only**	<1.20	1.21	1.71	3.52
**+ Explants**	<1.20	<1.20	1.95	<1.20
**+ Explants/Mφ**	<1.20	<1.20	1.58	1.58
**IL-10**				
	**4898**	**4836**	**2780**	**5178**
**T cells Only**	<1.09	1.675	1.18	1.77
**+ Explants**	2.95	3.44	3.96	2.74
**+ Explants/Mφ**	1.73	17.03	8.12	8.31
**Arginase**				
	**4898**	**4836**	**2780**	**5178**
**T cells Only**	3663.90	3923.90	2346.75	3975.41
**+ Explants**	3052.86	3285.92	2346.46	2819.79
**+ Explants/Mφ**	5712.62	4302.11	4212.98	5670.56

## Data Availability

Data is contained within the article or Supplementary Materials. The corresponding authors can provide raw data of results upon reasonable request.

## Supplementary Materials

The following are available online at https://www.mdpi.com/2227-9059/9/3/302/s1, Table S1, Table S2, Figure S1, Table S3.

## Abbreviations

AGFB	AG02603 fibroblasts
CAFs	Cancer-associated fibroblasts
EBV	Epstein-Barr virus
FBS	Fetal bovine serum
HCMV	Human cytomegalovirus
IHC	Immunohistochemistry
iNOS	Nitric oxide synthase
LPS	Lipopolysaccharide
Mφ	Monocyte-derived macrophages
MDSCs	Myeloid-derived suppressor cells
MFI	Mean fluorescence intensity
MT	Transitional myeloid cells
MU	Unpolarised myeloid cells
NSCLC	Non-small cell lung cancer
PBMCs	Peripheral blood mononuclear cells
PBS	Phosphate-buffered saline
RT	Room temperature
TAM	Tumour-associated macrophage
TGFβ1	Transforming growth factor β1
TME	Tumour microenvironment
TT	Tetanus toxoid
VP	Viral peptide

## References

[1] Gordon S. (2007). The macrophage: Past, present and future. Eur. J. Immunol..

[2] Genin M., Clement F., Fattaccioli A., Raes M., Michiels C. (2015). M1 and M2 macrophages derived from THP-1 cells differentially modulate the response of cancer cells to etoposide. BMC Cancer.

[3] Takahashi H., Sakakura K., Kudo T., Toyoda M., Kaira K., Oyama T., Chikamatsu K. (2017). Cancer-associated fibroblasts promote an immunosuppressive microenvironment through the induction and accumulation of protumoral macrophages. Oncotarget.

[4] Genard G., Lucas S., Michiels C. (2017). Reprogramming of Tumor-Associated Macrophages with Anticancer Therapies: Radiotherapy versus Chemo- and Immunotherapies. Front. Immunol.

[5] Mantovani A., Sica A., Sozzani S., Allavena P., Vecchi A., Locati M. (2004). The chemokine system in diverse forms of macrophage activation and polarization. Trends Immunol..

[6] Yuan A., Hsiao Y.J., Chen H.Y., Chen H.W., Ho C.C., Chen Y.Y., Liu Y.C., Hong T.H., Yu S.L., Chen J.J. (2015). Opposite Effects of M1 and M2 Macrophage Subtypes on Lung Cancer Progression. Sci Rep..

[7] Zhang B., Yao G., Zhang Y., Gao J., Yang B., Rao Z. (2011). M2-Polarized tumor-associated macrophages are associated with poor prognoses resulting from accelerated lymphangiogenesis in lung adenocarcinoma. Clinics (Sao Paulo).

[8] Chen J.J., Lin Y.C., Yao P.L., Yuan A., Chen H.Y., Shun C.T., Tsai M.F., Chen C.H., Yang P.C. (2005). Tumor-associated macrophages: The double-edged sword in cancer progression. J. Clin. Oncol..

[9] Yusen W., Xia W., Shengjun Y., Shaohui Z., Hongzhen Z. (2018). The expression and significance of tumor associated macrophages and CXCR4 in non-small cell lung cancer. J. Buon.

[10] Sumitomo R., Hirai T., Fujita M., Murakami H., Otake Y., Huang C. (2019). M2 Tumor-Associated Macrophages Promote Tumor Progression in Non-Small-Cell Lung Cancer. Exp. Ther. Med..

[11] Johnson D.B., Rioth M.J., Horn L. (2014). Immune Checkpoint Inhibitors in NSCLC. Curr. Treat. Options Oncol.

[12] Karekla E., Liao W.J., Sharp B., Pugh J., Reid H., Quesne J.L., Moore D., Pritchard C., MacFarlane M., Pringle J.H. (2017). Ex Vivo Explant Cultures of Non-Small Cell Lung Carcinoma Enable Evaluation of Primary Tumor Responses to Anticancer Therapy. Cancer Res..

[13] Ishiguro T., Ohata H., Sato A., Yamawaki K., Enomoto T., Okamoto K. (2017). Tumor-derived spheroids: Relevance to cancer stem cells and clinical applications. Cancer Sci..

[14] Sanmamed M.F., Chester C., Melero I., Kohrt H. (2016). Defining the optimal murine models to investigate immune checkpoint blockers and their combination with other immunotherapies. Ann. Oncol.

[15] Rebelo S.P., Pinto C., Martins T.R., Harrer N., Estrada M.F., Loza-Alvarez P., Cabeçadas J., Alves P.M., Gualda E.J., Sommergruber W. (2018). 3D-3-culture: A tool to unveil macrophage plasticity in the tumour microenvironment. Biomaterials.

[16] Kuen J., Darowski D., Kluge T., Majety M. (2017). Pancreatic cancer cell/fibroblast co-culture induces M2 like macrophages that influence therapeutic response in a 3D model. PLoS ONE.

[17] Tevis K.M., Cecchi R.J., Colson Y.L., Grinstaff M.W. (2017). Mimicking the Tumor Microenvironment to Regulate Macrophage Phenotype and Assessing Chemotherapeutic Efficacy in Embedded Cancer Cell/Macrophage Spheroid Models. Acta Biomater.

[18] Raghavan S., Mehta P., Xie Y., Lei Y.L., Mehta G. (2019). Ovarian cancer stem cells and macrophages reciprocally interact through the WNT pathway to promote pro-tumoral and malignant phenotypes in 3D engineered microenvironments. J. Immunother Cancer.

[19] Schmid J.O., Dong M., Haubeiss S., Friedel G., Bode S., Grabner A., Ott G., Murdter T.E., Oren M., Aulitzky W.E. (2012). Cancer cells cue the p53 response of cancer-associated fibroblasts to cisplatin. Cancer Res..

[20] Weiswald L.B., Bellet D., Dangles-Marie V. (2015). Spherical cancer models in tumor biology. Neoplasia.

[21] Chandorkar P., Posch W., Zaderer V., Blatzer M., Steger M., Ammann C.G., Binder U., Hermann M., Hortnagl P., Lass-Florl C. (2017). Fast-track development of an in vitro 3D lung/immune cell model to study Aspergillus infections. Sci Rep..

[22] Lang D.S., Droemann D., Schultz H., Branscheid D., Martin C., Ressmeyer A.R., Zabel P., Vollmer E., Goldmann T. (2007). A novel human ex vivo model for the analysis of molecular events during lung cancer chemotherapy. Respir Res..

[23] Parry-Jones A., Spary L. (2018). The Wales Cancer Bank (WCB). Open J. Bioresour..

[24] Maritan S., Lian E., Mulligan L. (2017). An Efficient and Flexible Cell Aggregation Method for 3D Spheroid Production. J. Vis. Exp. JoVE.

[25] Ryu N., Lee S., Park H. (2019). Spheroid Culture System Methods and Applications for Mesenchymal Stem Cells. Cells.

[26] Coleman S., Clayton A., Mason M.D., Jasani B., Adams M., Tabi Z. (2005). Recovery of CD8+ T-cell function during systemic chemotherapy in advanced ovarian cancer. Cancer Res..

[27] Currier J., Kuta E., Turk E., Earhart L., Loomis-Price L., Janetzki S., Ferrari G., Birx D., Cox J. (2002). A panel of MHC class I restricted viral peptides for use as a quality control for vaccine trial ELISPOT assays. J. Immunol. Methods.

[28] Clappaert E.J., Murgaski A., Van Damme H., Kiss M., Laoui D. (2018). Diamonds in the Rough: Harnessing Tumor-Associated Myeloid Cells for Cancer Therapy. Front. Immunol.

[29] Sica A., Porta C., Morlacchi S., Banfi S., Strauss L., Rimoldi M., Totaro M.G., Riboldi E. (2012). Origin and Functions of Tumor-Associated Myeloid Cells (TAMCs). Cancer Microenviron..

[30] Solinas G., Germano G., Mantovani A., Allavena P. (2009). Tumor-associated macrophages (TAM) as major players of the cancer-related inflammation. J. Leukoc Biol..

[31] Wynn T.A., Barron L. (2010). Macrophages: Master Regulators of Inflammation and Fibrosis. Semin. Liver Dis..

[32] Fairweather D., Cihakova D. (2009). Alternatively activated macrophages in infection and autoimmunity. J. Autoimmun..

[33] Spary L.K., Salimu J., Webber J.P., Clayton A., Mason M.D., Tabi Z. (2014). Tumor stroma-derived factors skew monocyte to dendritic cell differentiation toward a suppressive CD14(+) PD-L1(+) phenotype in prostate cancer. Oncoimmunology.

[34] Balestrini J.L., Chaudhry S., Sarrazy V., Koehler A., Hinz B. (2012). The mechanical memory of lung myofibroblasts. Integr. Biol. (Camb).

[35] Hinz B. (2010). The myofibroblast: Paradigm for a mechanically active cell. J. Biomech..

[36] Webber J., Steadman R., Mason M.D., Tabi Z., Clayton A. (2010). Cancer exosomes trigger fibroblast to myofibroblast differentiation. Cancer Res..

[37] Webber J.P., Spary L.K., Sanders A.J., Chowdhury R., Jiang W.G., Steadman R., Wymant J., Jones A.T., Kynaston H., Mason M.D. (2015). Differentiation of tumour-promoting stromal myofibroblasts by cancer exosomes. Oncogene.

[38] Menzies F.M., Henriquez F.L., Alexander J., Roberts C.W. (2010). Sequential expression of macrophage anti-microbial/inflammatory and wound healing markers following innate, alternative and classical activation. Clin. Exp. Immunol..

[39] Mantovani A., Sozzani S., Locati M., Allavena P., Sica A. (2002). Macrophage polarization: Tumor-associated macrophages as a paradigm for polarized M2 mononuclear phagocytes. Trends Immunol..

[40] Mills C.D., Thomas A.C., Lenz L.L., Munder M. (2014). Macrophage: SHIP of Immunity. Front. Immunol..

[41] Aras S., Zaidi M.R. (2017). TAMeless traitors: Macrophages in cancer progression and metastasis. Br. J. Cancer.

[42] Buechler C., Ritter M., Orso E., Langmann T., Klucken J., Schmitz G. (2000). Regulation of scavenger receptor CD163 expression in human monocytes and macrophages by pro- and antiinflammatory stimuli. J. Leukoc Biol..

[43] Almatroodi S.A., McDonald C.F., Darby I.A., Pouniotis D.S. (2016). Characterization of M1/M2 Tumour-Associated Macrophages (TAMs) and Th1/Th2 Cytokine Profiles in Patients with NSCLC. Cancer Microenviron..

[44] Jackute J., Zemaitis M., Pranys D., Sitkauskiene B., Miliauskas S., Vaitkiene S., Sakalauskas R. (2018). Distribution of M1 and M2 macrophages in tumor islets and stroma in relation to prognosis of non-small cell lung cancer. BMC Immunol..

[45] Maniecki M.B., Etzerodt A., Moestrup S.K., Moller H.J., Graversen J.H. (2011). Comparative assessment of the recognition of domain-specific CD163 monoclonal antibodies in human monocytes explains wide discrepancy in reported levels of cellular surface CD163 expression. Immunobiology.

[46] Tarique A.A., Logan J., Thomas E., Holt P.G., Sly P.D., Fantino E. (2015). Phenotypic, functional, and plasticity features of classical and alternatively activated human macrophages. Am. J. Respir Cell Mol. Biol..

[47] Ambarus C.A., Krausz S., van Eijk M., Hamann J., Radstake T.R., Reedquist K.A., Tak P.P., Baeten D.L. (2012). Systematic validation of specific phenotypic markers for in vitro polarized human macrophages. J. Immunol. Methods.

[48] Comito G., Segura C.P., Taddei M.L., Lanciotti M., Serni S., Morandi A., Chiarugi P., Giannoni E. (2017). Zoledronic acid impairs stromal reactivity by inhibiting M2-macrophages polarization and prostate cancer-associated fibroblasts. Oncotarget.

[49] Gajewski T.F., Fuertes M., Spaapen R., Zheng Y., Kline J. (2011). Molecular profiling to identify relevant immune resistance mechanisms in the tumor microenvironment. Curr. Opin. Immunol..

[50] Wu X., Peng M., Huang B., Zhang H., Wang H., Xue Z., Zhang L., Da Y., Yang D., Yao Z. (2013). Immune microenvironment profiles of tumor immune equilibrium and immune escape states of mouse sarcoma. Cancer Lett..

