# Supplementary material for: Macrophage Plasticity and Function in the Lung Tumour Microenvironment Revealed in 3D Heterotypic Spheroid and Explant Models
Source: Biomedicines. 2021 Mar 15;9(3):302. doi: 10.3390/biomedicines9030302 (PMC7999110; doi:10.3390/biomedicines9030302)
Supplement: Supplementary file 1 [file biomedicines-09-00302-s001.pdf]

Supplementary Materials

Macrophage plasticity and function in the lung tumour microenvironment revealed in 3D heterotypic spheroid and explant models

Authors
Lauren Evans[1*], Kate Milward[1*], Richard Attanoos[2,3], Aled Clayton[1], Rachel Errington[1], Zsuzsanna Tabi[1]

Affiliations
[1] Tissue MicroEnvironment Group, Division of Cancer and Genetics, School of Medicine, Cardiff University, Tenovus Building, University Hospital of Wales, Heath Park, Cardiff, CF14 4XN, UK.

[2] School of Medicine, Cardiff University, University Hospital of Wales, Heath Park, Cardiff, CF14 4XN, UK.

[3] Department of Cellular Pathology, University Hospital of Wales, Heath Park, Cardiff, CF14 4XN, UK.

*Correspondence: Lauren.evans@crick.ac.uk (L.E.); Tel.: +44 (0) 29206 87342; Milwardk@cardiff.ac.uk (K.M.); Tel.: +44 (0) 29206 87342

TABLE S1. The mean fluorescence intensity (MFI, A.U.) values for CD64, CD163, and CD206 for each arm of the NSCLC Mϕ polarisation experiments. Table shows the mean marker expression and standard error of the mean (SEM) values for all independent experiments performed in the (**A**) multi-component spheroid model and (**B**) patient explant model.

A

n = 3	MFI (A.U.)					
	CD64		CD163		CD206	
	Mean	SEM	Mean	SEM	Mean	SEM
**T = 0**	7770.5	4067.5	348.6	385.6	289.4	115.1
**H522**	233.5	117.6	210.2	46.7	5777.8	2287.3
**AGFG**	381.2	89.4	389.8	91.9	10875.0	4186.5
**H522/AGFB**	305.0	121.5	220.1	245.4	5582.6	2008.9
**M1**	4782.8	645.4	101.7	133.4	1545.1	1144.6
**M2**	334.1	42.1	1799.8	1139.9	24820.0	10777.0
**Media**	520.1	165.3	1785.5	1118.7	3384.3	1593.7

B

n = 5	MFI (A.U.)					
	CD64		CD163		CD206	
	Mean	SEM	Mean	SEM	Mean	SEM
**T = 0**	1008.8	225.7	1170.2	427.2	119.7	147.4
**Explants (TAMs)**	−878.7	1825.9	1175.5	709.2	1130.5	583.7
**Explants/Mϕ**	438.8	406.0	−349.8	712.9	1576.0	778.0
**M1**	2911.9	970.5	310.0	413.7	353.8	214.0
**M2**	844.7	346.8	356.0	319.5	715.0	223.0
**Media**	362.3	430.6	335.1	309.9	267.2	154.0

TABLE S2. The epitope sequence of each antigen used in the T cell suppression assays are shown, alongside the pathogen source and antigen reference for each peptide. The Immune Epitope Database (IEDB) was used to predict epitope sequences for the most common HLA (MHC-I and MHC-II) alleles from viral (or bacterial; TT) antigens. Epstein-Barr virus, EBV; Human cytomegalovirus, HCMV; Tetanus toxoid, TT.

**HLA restriction**	**Epitope sequence**	**Antigen reference**
A*01:01	CTELKLSDY	Influenza A (PR8) NP 44-52
A*01:01	VSDGGPNLY	Influenza A PB1 591-599
A*02:01	GILGFVFTL	Influenza A MP 58-66
A*02:01	NLVPMVATV	HCMV pp65 495-504
A*02:01	FLYALALLL	EBV LMP-2 356-364
A*03:01	ILRGSVAHK	Influenza A (PR8) NP 265-274
A*03:01	RLRAEAQVK	EBV EBNA 3A 603–611
A*11:01	ATIGTAMYK	EBV BRLF1 134-142
A*33; A*50	LQHYREVAAAK	EBV BZLF1 197-208
B*07:02	QPEWFRNVL	Influenza A PB1 329-337
B*07:02	QPRAPIRPI	EBV EBNA-3C 881-889
B*07:02	TPRVTGGGAM	HCMV pp65 418-427
B*08:01	RAKFKQLL	EBV BZLF1 190-197
B*08:01	QAKWRLQTL	EBV EBNA-3A 158-166
B44	EFFWDANDIY	HCMV pp65 512-521
B*44:05	EENLLDFVRF	EBV EBNA 3C 281-290
DRB1*01:01	TSLYNLRRGTALA	EBV EBNA1 15-527
DRB1*07:01	EPDVYYTSAFVFPTK	HCMV pp65 177-191
DRB1*15:01	MSIYVYALPLKMLNI	HCMV pp65 109-123
DRB1*04:01	AEGLRALLARSHVER	EBV EBNA1 482-496
DRB1*01:01	PKYVKQNTLKLAT	Influenza A HA1 307-319
DRB1*07:01	PDDYSNTHSTRYVTV	HCMV gB 215-229
DRB1*11:01	VSIDKFRIFCKALNPK	TT 1084-1099
MULTIPLE	AAFEDLRVLSFIKGTK	Influenza A NP 336-351

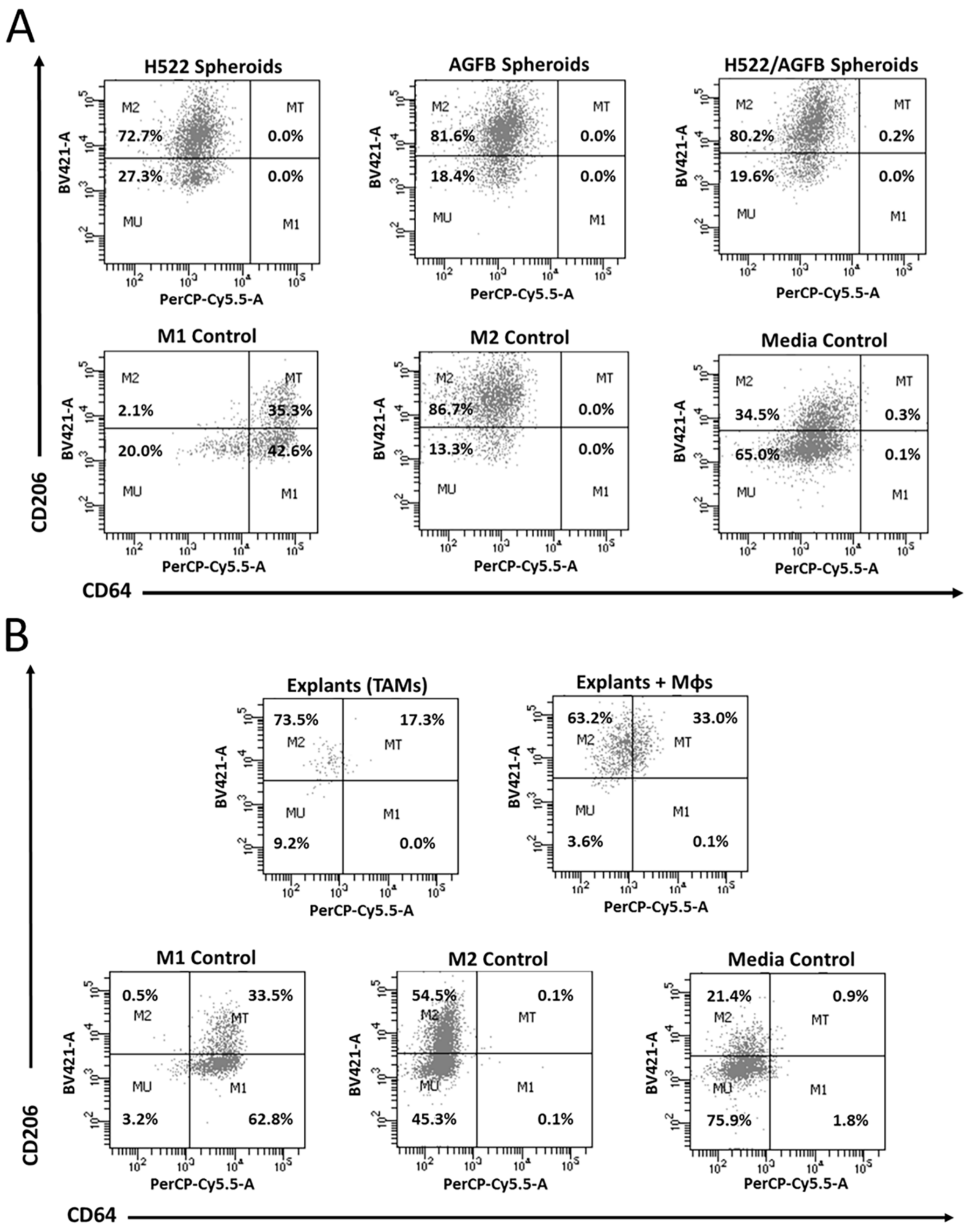

FIGURE S1. $CD206^{hi/lo}CD64^{hi/lo}$ quadrant (two-marker) flow cytometry gating denoting unpolarised myeloid cells (MU), M1-like Mϕs, M2-like Mϕs, and transitional myeloid cell (MT) populations for all culture conditions in the (**A**) spheroid- and (**B**) explant-mediated Mϕ polarisation experiments. Two-marker analysis results are given as a percentage of parent (live CD14^{+}) cells.

TABLE S3. (**A**) The mean fluorescence intensity (MFI, A.U.) values for IL-12p70, IL-12p40, IL-6, IL-1β, IL-23, CXCL10, IL-4, CCL17, and IL-1RA for each arm of the explant-mediated soluble factor secretion experiments. Table shows the mean MFI and standard error of the mean (SEM) values for each of the 4 patient (4898, 4836, 2780, and 5178) experiments. (**B**) The mean concentration (pg/ml) of each target present in T cell co-culture supernatants was determined using individual protein standards for each of the 4 patient experiments.

A

**Cytokine/chemokine MFI (A.U.)**					
**IL-12p70**					
	**4898**	**4836**	**2780**	**5178**	**Mean ± SEM**
**T cells Only**	40.54	56.96	46.70	55.94	50.03 ± 3.92
**+ Explants**	40.54	64.15	102.12	50.29	64.27 ± 13.51
**+ Explants/Mϕ**	44.65	52.86	53.37	83.13	58.50 ± 8.45
**IL-12p40**					
	**4898**	**4836**	**2780**	**5178**	**Mean ± SEM**
**T cells Only**	52.34	54.39	51.31	79.54	39.39 ± 6.74
**+ Explants**	47.73	56.45	54.40	55.42	53.50 ± 1.97
**+ Explants/Mϕ**	52.86	55.94	51.31	70.82	57.73 ± 4.47
**IL-6**					
	**4898**	**4836**	**2780**	**5178**	**Mean ± SEM**
**T cells Only**	5711.50	15422.50	4286.00	17757.00	10794.25 ± 3392.30
**+ Explants**	95768.50	95570.00	104726.00	98811.00	98718.88 ± 2135.30
**+ Explants/Mϕ**	63339.00	98066.00	97617.50	99214.00	89559.13 ± 8746.50
**IL-1β**					
	**4898**	**4836**	**2780**	**5178**	**Mean ± SEM**
**T cells Only**	61590.00	61730.00	55116.50	88211.00	66661.88 ± 7346.81
**+ Explants**	71586.00	66539.50	79306.00	88200.50	76408 ± 4579.07
**+ Explants/Mϕ**	69331.00	74208.00	79261.00	90690.50	78372.63 ± 4579.07
**IL-23**					
	**4898**	**4836**	**2780**	**5178**	**Mean ± SEM**
**T cells Only**	84.16	81.59	83.13	87.24	84.03 ± 1.19
**+ Explants**	80.56	88.78	84.67	84.67	84.67 ± 1.68
**+ Explants/Mϕ**	87.75	85.70	84.16	92.37	87.49 ± 1.78

CXCL10					
	**4898**	**4836**	**2780**	**5178**	**Mean ± SEM**
**T cells Only**	793.34	18103.00	1643.50	15462.50	9000.58 ± 4,28.58
**+ Explants**	12589.00	37647.50	88606.50	7113.50	36489.13 ± 18600.42
**+ Explants/Mϕ**	967.89	45726.50	106449.00	60880.00	53505.85 ± 21752.61
**IL-4**					
	**4898**	**4836**	**2780**	**5178**	**Mean ± SEM**
**T cells Only**	192.43	198.08	223.22	204.23	204.49 ± 6.69
**+ Explants**	198.59	196.54	1,327.83	222.71	486.41 ± 280.53
**+ Explants/Mϕ**	194.48	206.29	222.71	207.83	207.82 ± 5.79
**CCL17**					
	**4898**	**4836**	**2780**	**5178**	**Mean ± SEM**
**T cells Only**	98.01	1019.26	129.83	478.77	431.47 ± 214.07
**+ Explants**	546.50	1970.00	7159.00	912.61	2647.03 ± 1533.97
**+ Explants/Mϕ**	138.04	3184.50	1563.73	1,534.00	1605.07 ± 622.73
**IL-1RA**					
	**4898**	**4836**	**2780**	**5178**	**Mean ± SEM**
**T cells Only**	160.11	340.73	112.90	321.23	233.74 ± 57.10
**+ Explants**	2030.50	6615.50	2855.00	1435.26	3234.06 ± 1164.12
**+ Explants/Mϕ**	2674.58	7359.00	6259.50	2510.50	4700.89 ± 1238.23

B

Cytokines/chemokine concentration (pg/ml)				
**IL-12p70**				
	**4898**	**4836**	**2780**	**5178**
**T cells Only**	<0.50	<0.50	<0.50	<0.50
**+ Explants**	<0.50	<0.50	0.64	<0.50
**+ Explants/Mϕ**	<0.50	<0.50	<0.50	<0.50
**IL-12p40**				
	**4898**	**4836**	**2780**	**5178**

**T cells Only**	<0.94	<0.94	<0.94	1.62
**+ Explants**	<0.94	<0.94	<0.94	<0.94
**+ Explants/Mϕ**	<0.94	<0.94	<0.94	1.10
**IL-6**				
	**4898**	**4836**	**2780**	**5178**
**T cells Only**	56.70	301.35	33.67	398.26
**+ Explants**	17636.64	17525.41	23237.59	19318.45
**+ Explants/Mϕ**	9692.70	18887.90	18673.50	19562.94
**IL-1β**				
	**4898**	**4836**	**2780**	**5178**
**T cells Only**	4306.78	4281.39	3174.85	16134.15
**+ Explants**	6814.84	5440.14	9906.45	16140.48
**+ Explants/Mϕ**	6126.78	7803.59	10094.08	18697.51
**IL-23**				
	**4898**	**4836**	**2780**	**5178**
**T cells Only**	<0.66	<0.66	<0.66	<0.66
**+ Explants**	<0.66	0.69	<0.66	<0.66
**+ Explants/Mϕ**	0.69	<0.66	<0.66	1.12
**CXCL10**				
	**4898**	**4836**	**2780**	**5178**
**T cells Only**	7.99	175.87	17.26	150.02
**+ Explants**	124.65	570.79	1890.04	69.01
**+ Explants/Mϕ**	9.98	577.67	3042.40	870.18
**IL-4**				
	**4898**	**4836**	**2780**	**5178**
**T cells Only**	<1.09	<1.09	1.12	<1.09
**+ Explants**	<1.09	<1.09	48.8	1.44
**+ Explants/Mϕ**	<1.09	<1.09	1.23	<1.09
**CCL17**				

	**4898**	**4836**	**2780**	**5178**
**T cells Only**	2.63	54.78	2.88	23.64
**+ Explants**	27.39	103.56	398.56	48.91
**+ Explants/Mϕ**	6.77	162.76	81.40	81.81
**IL-1RA**				
	**4898**	**4836**	**2780**	**5178**
**T cells Only**	<2.22	2.60	<2.22	3.10
**+ Explants**	9.63	27.91	11.98	7.48
**+ Explants/Mϕ**	10.89	35.48	23.52	11.08
